# Development and Validation of a Flow-Dependent Endothelialized 3D Model of Intracranial Atherosclerotic Disease

**DOI:** 10.1007/s12975-024-01310-4

**Published:** 2024-11-14

**Authors:** Grace Prochilo, Chuanlong Li, Eleni Miliotou, Russell Nakasone, Alissa Pfeffer, Charles Beaman, Naoki Kaneko, David S. Liebeskind, Jason D. Hinman

**Affiliations:** 1 Department of Neurology, David Geffen School of Medicine, Gordon Neuroscience Research Building, The University of California, 635 Charles E. Young Dr. South, Room 415, Los AngelesLos Angeles, CA USA; 2 Department Radiology, David Geffen School of Medicine, The University of California, Los Angeles, Los Angeles, CA USA; 3 Department of Neurology, Department of Veterans Affairs Medical Center, Los Angeles, CA USA

**Keywords:** Stroke, Intracranial atherosclerosis, Endothelia, Blood flow, Computational fluid dynamics

## Abstract

Intracranial atherosclerotic disease (ICAD) is a major cause of stroke globally, with mechanisms presumed to be shared with atherosclerosis in other vascular regions. Due to the scarcity of relevant animal models, testing biological hypotheses specific to ICAD is challenging. We developed a workflow to create patient-specific models of the middle cerebral artery (MCA) from neuroimaging studies, such as CT angiography. These models, which can be endothelialized with human endothelial cells and subjected to flow forces, provide a reproducible ICAD model. Using imaging from the SAMMPRIS clinical trial, we validated this novel model. Computational fluid dynamics flow velocities correlated strongly with particle-derived flow, regardless of stenosis degree. Post-stenotic flow disruption varied with stenosis severity. Single-cell RNA-seq identified flow-dependent endothelial gene expression and specific endothelial subclusters in diseased MCA segments, including upregulated genes linked to atherosclerosis. Confocal microscopy revealed flow-dependent changes in endothelial cell proliferation and morphology in vessel segments related to stenosis. This platform, rooted in the specific anatomy of cerebral circulation, enables detailed modeling of ICAD lesions and pathways. Given the high stroke risk associated with ICAD and the lack of effective treatments, these experimental models are crucial for developing new ICAD-related stroke therapies.

## Introduction

Intracranial atherosclerosis disease (ICAD) is a highly prevalent chronic disease that is a leading cause of stroke worldwide [1, 2]. Mechanical stenting of ICAD lesions has repeatedly failed clinical trials [3–5], indicating that mechanical disruption of lesions is insufficient to reduce stroke risk. Despite its high prevalence and causative role in ischemic stroke, the pathophysiologic mechanisms of ICAD are incompletely understood. Parallels to atherosclerosis in other circulatory systems may be relevant. However, drawing these parallels makes several assumptions that disregard the distinct anatomy and rates of blood flow in the proximal cerebral vessels that create a unique inter-relationship between the biophysical forces of flow and the endothelial surface in ICAD lesions. This is clear when considering the differential patterns of recurrent stroke in ICAD, including distal cerebral hypoperfusion accounting for 22.7% of stroke recurrence as indicated by a border zone infarct pattern while distal thromboembolic strokes account for 27.3% as indicated by a cortical or territorial pattern [6]. Moreover, the rates of ICAD progression and impact of the degree of stenosis do not drive rates of ischemic events as in the coronary or carotid circulations [7, 8]. Therefore, the development of experimental models of ICAD that can recapitulate the anatomic features of human ICAD lesions with the flow characteristics driven by the lesion and endothelial cell biology can inform the underlying vascular biology that may advance this field beyond stenting.

Various models animal and in vitro models of atherosclerosis exist [9]. All existing models have limitations that range from the lack of characteristic atherosclerotic plaques in rodent models to the use of artificial stenoses in vitro that do not mimic the anatomic features of ICAD lesions seen in patients. We previously developed an endothelialized 3D flow model of intracranial stenosis using patient-derived angiographic imaging and used it to identify the flow-responsive nature of angiotensin-converting enzyme 2 (ACE2) in the context of binding to Sars-CoV2 spike protein [10]. While this model provides a unique platform to study how stenosis-related flow forces drive focal changes in endothelial biology, the selection of patient-derived images as a source for the model can be biased. To establish a true representative experimental model of ICAD, case selection should include lesions that have a proven role in driving stroke. This has been a key limitation of animal models of ICAD that often develop fatty deposits and, in some cases, characteristic atherosclerotic lesions but infrequently develop localized thrombosis or embolism leading to stroke, as in ICAD patients [9]. The Stenting and Aggressive Medical Management for Preventing Recurrent Stroke in Intracranial Stenosis (SAMMPRIS) trial included patients with symptomatic intracranial atherosclerosis and tested the role of mechanical stent placement to reduce stroke risk [3]. The design of SAMMPRIS labels ICAD lesions as symptomatic and, when considering the MCA, enables a within-subject comparison vessel that is asymptomatic regardless of whether it harbors a stenosis. We sought to take advantage of the SAMMPRIS imaging dataset to determine if our endothelialized 3D flow model can represent flow features of ICAD as suggested by computational fluid dynamics (CFD) and expand the biologic measures available in this model system.

Here, we show the successful development and validation of an experimental model of intracranial atherosclerosis derived from the definitive clinical trial in ICAD. In MCA segments from SAMMPRIS, we mapped CFD flow profiles and experimentally validated these features in vitro*.* Additionally, we demonstrate the applicability of single-cell RNA-sequencing (scRNA-seq) as well as confocal microscopy to illustrate the unique endothelial cell biology driven by ICAD-related flow disruptions in this 3D flow system. While we sought here only to introduce and validate this model of ICAD, this powerful approach may be used across the variety of ICAD lesions to establish novel biophysical relationships between flow and endothelial biology relevant to stroke risk due to ICAD.

## Methodology

### Source Imaging

All source imaging was derived from the SAMMPRIS trial [3]. Baseline computed tomography angiography (CTA) images from the aggressive medical management (MM) and aggressive medical management plus percutaneous transluminal angioplasty and stenting (PTAS) groups were used for model generation. The present analyses focused only on MCA cases to have a within-subjects internal control. Specific cases were then excluded due to excessive tortuosity, the presence of multiple symptomatic lesions within one case, and/or proximity of the lesion to a bifurcation.

### PDMS Model Generation

Digital Imaging and Communication in Medicine (DICOM) format imaging data were acquired from CTA and converted to a stereolithography format (.stl). For each case, the diseased MCA (dMCA) with the symptomatic lesion and the normal MCA (nMCA) on the contralateral side were segmented from a larger cerebrovascular network, and straight artificial tubes of uniform diameter were artificially attached to the inlet and outlet sides of each MCA segment to facilitate perfusion culture. For each segmented MCA model, the.stl file was run on a Mojo 3D printer (Stratasys, Eden Prairie, MN) to create a physical acrylonitrile butadiene styrene (ABS) vascular mold. The base and supports of the 3D-printed mold were manually removed after printing. The vascular mold was chemically smoothed in an acetone solvent to remove the stair-like layers of the printed filament. After drying, the mold was coated with degassed polydimethylsiloxanes (PDMS) and left to cure. The ABS mold was removed to produce a hollow PDMS model.

### Time-Lapse Particle Flow Studies

The PDMS vascular model was perfused with a viscosity-adjusted solution containing Fluorescent Orange Polyethylene Microspheres (1.00 g/cc, 45–53 um; Cospheric) and Dextran 200,000 (3.9 cP, Fujifilm Wako Chemicals) at a flow rate of 50 mL/min. Videos of particle flow were acquired on a Sony DSC-RX10 Mark4 Digital Camera at 960 fps. Videos were processed to optimize the signal-to-background ratio in FIJI. The Simple Linear Assignment Problem (LAP) Tracker in the FIJI TrackMate macro was used to track the path of individual particles over 400 frames (≈0.42 s). To ignore stationary particles and intermittent noise, particle tracks with a displacement of less than two pixels or a duration of less than three frames were excluded.

For the flow speed correlation with CFD velocity profiles, each datapoint represented the speed of a particle between two successive frames and its average location between frames along the long axis of the vessel model. The linking maximum distance was set to 200 pixels, the gap-closing maximum distance was set to 100 pixels, and the gap-closing max frame gap was set to 2 frames. For the quantification of disturbed flow in the post-stenotic region, each data point represented the linearity ratio of a given particle’s track over the length of the video. The linearity ratio is defined as the ratio of a particle’s net displacement over its total distance traveled. The linking maximum distance was set to 15 pixels, the gap-closing maximum distance was set to 15 pixels, and the gap-closing max frame gap was set to 2 frames.

### CFD Analysis

For each .stl MCA model, a volumetric mesh was generated, consisting of polyhedral cells in the center region and transitional layers near the arterial wall. After mesh generation, pulsatile flow conditions were utilized to intentionally simulate human physiologic conditions using FLUENT (Ansys Inc., Canonsburg, PA). Poiseuille flow calculations with a volumetric flow rate of 50 ml/min were used to generate velocity distributions for inflow conditions and reference standard boundary conditions are applied at the pressure outlet(s). Blood density and blood viscosity values used were 993 kg/m3 and 3.95 cP, respectively. The convergence of the simulation was verified by monitoring the continuity residual and achieved when the monitored value showed oscillations on the order of < 1 × 10^−10^. A double-precision solver was utilized for all models. A coupled scheme was used to couple velocity and pressure, and discretization was accurate to the second order. For velocity comparisons with particle flow studies, the velocity streamlines were collapsed onto a 2D plane. CFD outputs included velocity (m/s) and wall shear stress (dyn/cm^2^).

### Endothelialization and Perfusion Culture

The PDMS model and perfusion tubing were autoclaved for sterilization. To enhance hydrophilization, the PDMS model was soaked in 10% (3-aminopropyl) trimethoxysilane in 100% ethanol overnight and then washed with water. The model was immersed in 0.1 mmol/L sulfosuccinimidyl-6-(4′-azido-2′-ni-trophenylamino)-hexanoate (sulfo-SANPAH) in water and exposed to UV radiation (10 min × 2 treatments; 365 nm, 36 W, 5 cm distance) to activate cross-linking. The model was washed with PBS and immersed in a 40 µg/mL solution of human fibronectin in PBS. Human Umbilical Vein Endothelial Cells (HUVECs; Cell Systems, Inc.) between passages 6 and 10 were cultured in complete Endothelial Cell Growth Medium 2 (PromoCell) with 1% Penicillin–Streptomycin (Gibco) in a CO_2_ incubator (37 °C, 5% CO_2_) then seeded into the lumen of the model. To attempt to promote cell adhesion on all surfaces, the model was placed in a 3D rotating instrument at 1 rotation per minute for 48 h in a CO_2_ incubator. Following rotation culture, the inlet and outlet of the endothelialized model were connected to autoclaved silicone perfusion tubing, which were connected in series to a perfusion pump and bottle of complete media containing Dextran 200,000 (3.9 cP, Fujifilm Wako Chemicals) to match the viscosity of human blood. The model was perfused with viscosity-adjusted media for 48 h in a CO_2_ incubator.

### Generation of Single-Cell Suspension

Cell suspensions were generated from three sample conditions: a stenotic dMCA 3D biologic flow model, an asymptomatic nMCA 3D biologic flow model, and a static monolayer culture. Both 3D biologic flow models underwent endothelialization and perfusion culture for 48 h, and then flow was halted. The lumen of the model was gently washed with PBS. The model was filled with 0.4 mL TryplE (Gibco) and incubated at 37 °C with 5% CO2 for 10 min. Cells were then mechanically agitated with a syringe plunger to facilitate cell detachment. Subsequently, the model was washed twice with 0.4 mL complete media to neutralize the TryplE and collect the remaining cells into a tube. For the static culture, the cells were washed with 5 mL PBS, and then 3 mL TryplE was added to the T75 flask and incubated at 37 °C with 5% CO2 for 5 min. The sides of the flask were tapped gently to promote cell detachment, complete media was added, and the cell suspension was transferred to a tube. The resulting single-cell suspension for each condition was washed twice in PBS and then resuspended in 0.04% BSA in PBS. All three cell suspension samples were subjected to a cell viability quality control check before scRNA-seq.

### Single-Cell Sequencing and Informatics

ScRNA-seq using the 10X Genomics platform was executed by the UCLA Technology Center for Genomics & Bioinformatics. Single-cell cDNA and libraries were generated using a 10X Genomics Chromium 3’ GEX kit according to manufacturer protocol. Resulting cDNA, libraries, and sequencing results for all samples were subjected to standard quality control measures.

### Single-Cell Gene Expression Analysis

Three scRNA-seq datasets representing different experimental conditions (nMCA, dMCA, and static culture) were obtained as separate FASTQ files. The datasets were processed individually using the Seurat package (version 5) in R (version 4.2.2) for quality control and preprocessing. Raw sequencing reads were aligned to the reference genome GRCh38, followed by the removal of low-quality cells and mitochondrial genes. Cells with fewer than 200 genes or genes detected in fewer than 3 cells were considered low quality and filtered out. Additionally, cells with a high proportion of mitochondrial gene expression (greater than 10%) were also removed, as they may represent dying or stressed cells. Expression data were normalized using the “LogNormalize” method and scaled to 10,000 transcripts per cell. Highly variable genes were identified using the variance-stabilizing transformation (VST) method and scaled. After data preprocessing, dimensionality reduction and clustering were performed. Principal component analysis (PCA) identified the top 20 principal components (PCs). The PCA-reduced data was used to construct a shared nearest neighbor (SNN) graph. Clusters were identified with the FindClusters() function using the shared nearest neighbor modularity optimization with a clustering resolution set to 1.6 and employing the Louvain algorithm. After preprocessing each dataset individually, the datasets were merged, and batch effects were corrected. Dimensionality reduction and clustering were performed to identify distinct cell populations across the three conditions and between static and flow.

### Differential Expression Analysis

To identify marker genes specific to each subcluster, differential expression analysis was performed using the FindMarkers() function in Seurat. To identify genes differentially expressed between the three experimental conditions, first cells belonging to each condition were subset from the integrated Seurat object. To account for multiple hypothesis testing, the *p*-values obtained from the differential expression analysis were adjusted using the Benjamini–Hochberg correction. To gain insights into the biological processes and pathways associated with the differentially expressed genes, gene set enrichment analysis (GSEA) was performed. The results of differential expression analysis, including volcano plots, heatmaps, and gene expression profiles, were visualized using ggplot2, pheatmap, and patchwork. Lists of conserved cell markers for each subcluster were imported into GOrilla to identify enriched gene ontology (GO) terms. The resulting lists of GO terms and associated *p*-values for selected subclusters were imported into Revigo (v.1.8.1) to remove redundant GO terms using the following settings: medium list size (0.7), Homo Sapiens database, and SimRel semantic similarities.

### Cellular Morphology Labeling

After endothelialization and perfusion culture, the lumen of the model was washed with PBS. The model was filled with 2% paraformaldehyde for 10 min to fix the cells. After washing the lumen twice with PBS, the model was filled with 0.5% Triton X-100 in PBS for 5 min to permeabilize the cells. The cells were washed twice with PBS and then stained with rhodamine-phalloidin (Invitrogen, Waltham, MA, USA) and DAPI (Invitrogen, Waltham, MA, USA) for 1 h at room temperature.

### Image Acquisition

To prepare each PDMS model for imaging, the lumen was filled with PBS, plugged, and mounted on top of a microscope slide. Images were acquired on a Nikon AX R Confocal Microscope System (Nikon Instruments Inc., Melville, NY, U.S.A.) with a × 4 objective. To capture the full thickness of the curved luminal wall, z stacks of 10.3 µm were taken at a range of 360.5–484.1 µm.

### Quantification of Cell Morphology

Endothelial cell segmentation, quantification, and morphology in each image were determined using the CellProfiler program modified with the Cellpose plugin. Cellpose is a generalist, deep learning-based segmentation algorithm that is pre-trained to segment and identify a wide range of cell bodies, membranes, and nuclei as described previously [11]. The plugin provides a robust, accurate method for segmentation and is very corrigible by providing software for manual labeling or the ability to curate or incorporate our own training data. Filled masks are generated and quantified for each segmented cell or nuclei and morphological features such as area, eccentricity, and major/minor axis ratio can be automatically determined from each filled mask.

### Statistical Analyses

The degree of the flow speed correlation between the CFD and PDMS models was calculated using a Spearman Correlation. A simple linear regression was used to determine the best-fit lines for plots of speed correlation vs. percent stenosis, linearity ratio vs. percent stenosis, linearity ratio vs. nMCA distal x location, and linearity ratio vs. dMCA distal x location. A paired *t*-test was used to determine the statistical difference in linearity ratio between nMCA and dMCA groups. For the quantification of nuclei count, each datapoint represented the number of nuclei per quadrant in a given region (nMCA-prox, nMCA-dist, dMCA-prox, dMCA-dist); a Mann–Whitney test was used to determine statistical significance. For quantifications of cell area, cell minor/major axes ratio, and cell eccentricity, each datapoint represented the value for one cell, and the ROUT method (*Q* = 1) was used to identify outliers; a Kolmogorov–Smirnov test was used to determine statistical significance. The statistical analysis for the CFD-PDMS flow speed correlation was done using Python. All other statistical analysis was done using GraphPad Prism 10 software.

## Results

### Creation of a Patient-Specific, Endothelialized MCA ICAD Model

**Fig. 1:**
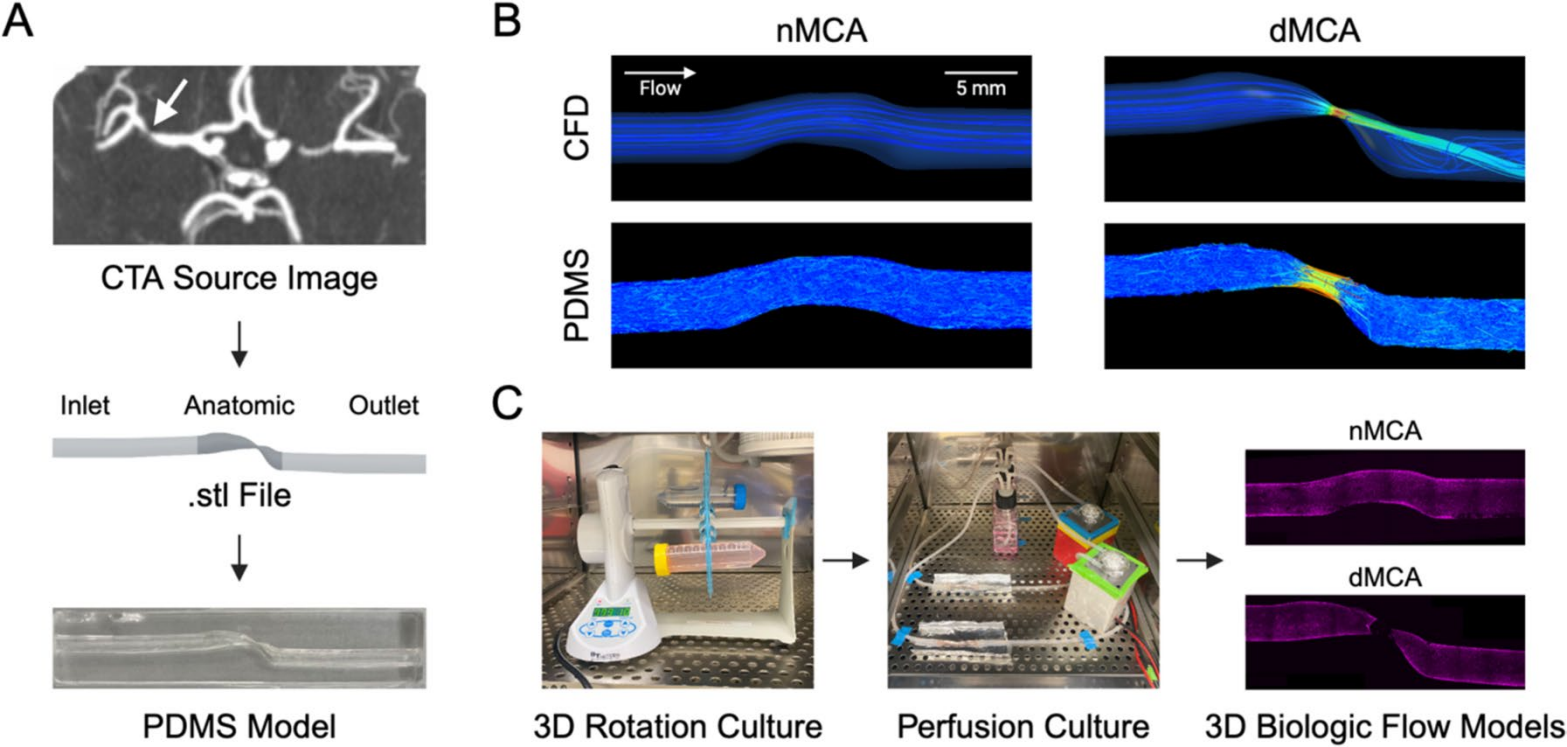
Creation of a patient-specific, endothelialized MCA ICAD model. **A** Representative modeling workflow from CTA source image to conversion to.stl file to generation of PDMS model. **B** Representative images of CFD velocity profiles and time-lapse particle flow studies on PDMS models generated from the same.stl files for one MCA case. **C** Representative modeling workflow from endothelial cell seeding in PDMS models and 3D rotation culture to perfusion culture to generate a pair of 3D biologic flow models for one MCA case

PDMS models of MCA arterial segments were generated from CTA imaging using intermediate.stl files and 3D-printed ABS vascular molds (Fig. 1A). In addition to stenotic dMCA segments, asymptomatic nMCA segments on the contralateral side were sub-selected as internal controls. We generated CFD velocity profiles from the.stl files for each MCA case. For validation against the CFD velocity profiles, we conducted time-lapse particle flow studies on PMDS models derived from the same.stl files using viscosity-adjusted fluid at a flow rate of 50 mL/min (Fig. 1B). To generate the complete 3D biologic flow model, we hydrophilized the PDMS model, coated it with human fibronectin, and seeded the lumen with a monolayer of human umbilical vein endothelial cells (HUVECs). We exposed the model to a 3D rotation culture for 48 h to promote uniform cell adhesion throughout the luminal surface. We perfused the model with viscosity-adjusted culture media (3.9 cPa) for an additional 48 h (Fig. 1C).

### Modeling of Symptomatic MCA ICAD Lesions from SAMMPRIS

**Fig. 2:**
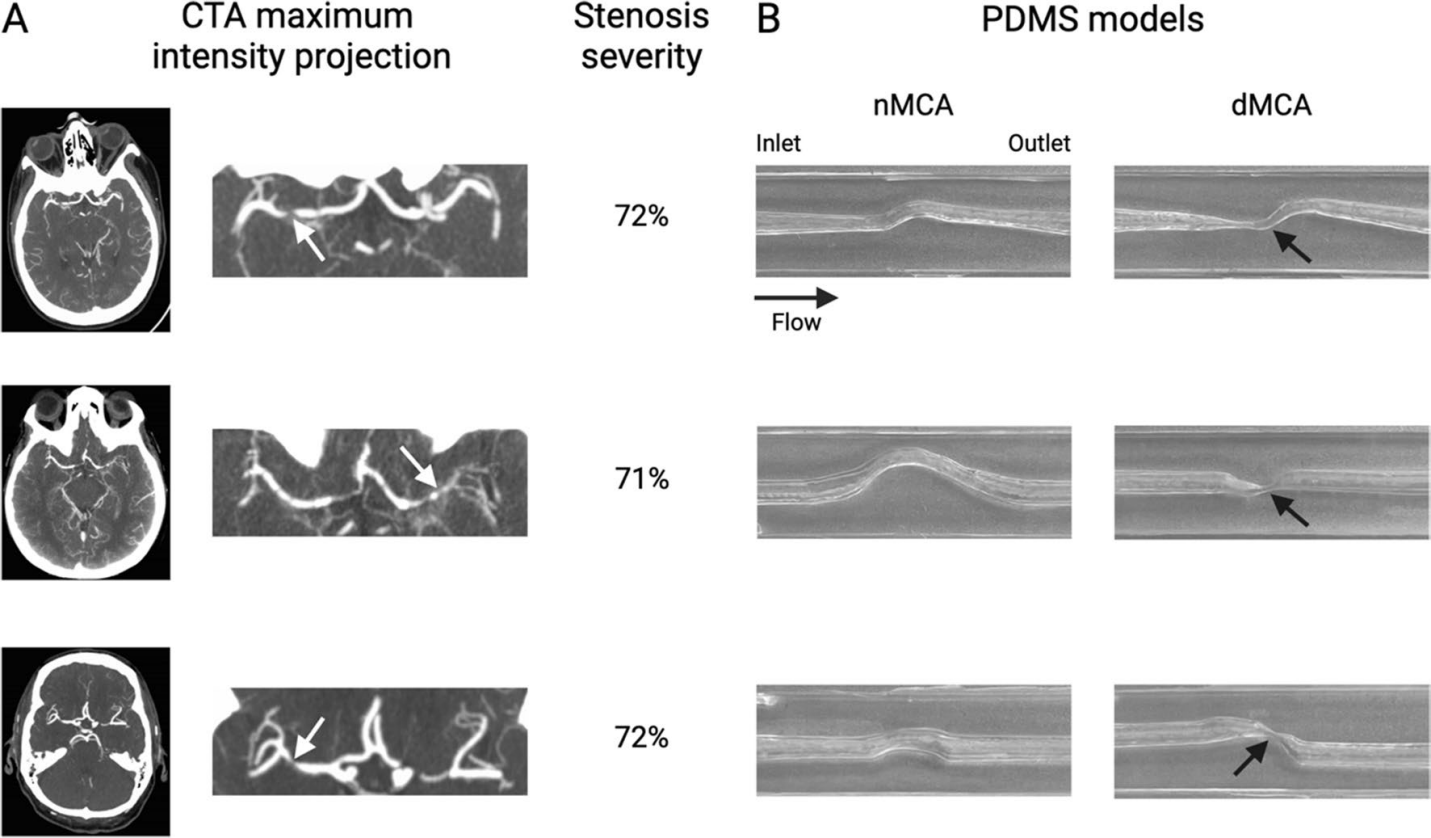
Modeling of symptomatic MCA ICAD lesions from SAMMPRIS. **A** Maximum intensity projections of CTA imaging for three representative MCA cases with associated stenosis percentages. White arrows indicate the location of stenosis. **B** normal MCA (nMCA) and diseased MCA (dMCA) PDMS models for the three adjacent MCA cases in **A**. Black arrows indicate the location of stenosis

Symptomatic MCA atherosclerotic lesions were sub-selected from SAMMPRIS CTA images (Table 1). Of all 451 cases enrolled in SAMMPRIS, only 140 cases (31.0%) had baseline CTA DICOM imaging at the time of trial enrollment. Among these, 50 subjects (11.1%) had MCA lesions. We included a total of eight MCA cases (1.7%), two cases from the MM group, and six cases from the PTAS group with an average rate of stenosis of 76.1%. There was considerable variance in stenosis length, stenosis diameter (76.1 ± 5.8%), and vessel tortuosity in the arterial segments included in this internally controlled analysis (Fig. 2A). Each dMCA model included 3–5 mm of pre-stenotic vessel and at least 2 mm of post-stenotic vessel along with the length of the stenosis itself, and each nMCA model included a similar total anatomical length (13.2 ± 4.5 mm) (Fig. 2B).

**Table 1:** SAMMPRIS dataset as source imaging for 3D cerebrovascular modeling

	Medical management (MM)	Percutaneous transluminal angioplasty and stenting (PTAS)	PDMS models generated
**Symptomatic qualifying artery**	No. of cases (% of cases)	No. of cases (% of cases)	No. of cases (no. of MM cases / no. of PTAS cases)
Internal carotid	49 (21.6)	45 (20.1)	N/A
Middle cerebral	105 (46.3)	92 (41.1)	8 (2 / 6)
Vertebral	22 (9.7)	38 (17.0)	N/A
Basilar	51 (22.5)	49 (21.9)	N/A
**Stenosis severity (%)**	No. of cases (% of cases)	No. of cases (% of cases)	No. of cases (no. of MM cases / no. of PTAS cases)
70–79	102 (44.9)	107 (48.0)	5 (2 / 3)
80–89	97 (42.3)	92 (41.3)	2 (0 / 3)
90–99	28 (12.3)	24 (10.8)	0 / 0

### Flow Patterns in 3D PDMS Models Mimic ICAD-Specific Hemodynamics

**Fig. 3:**
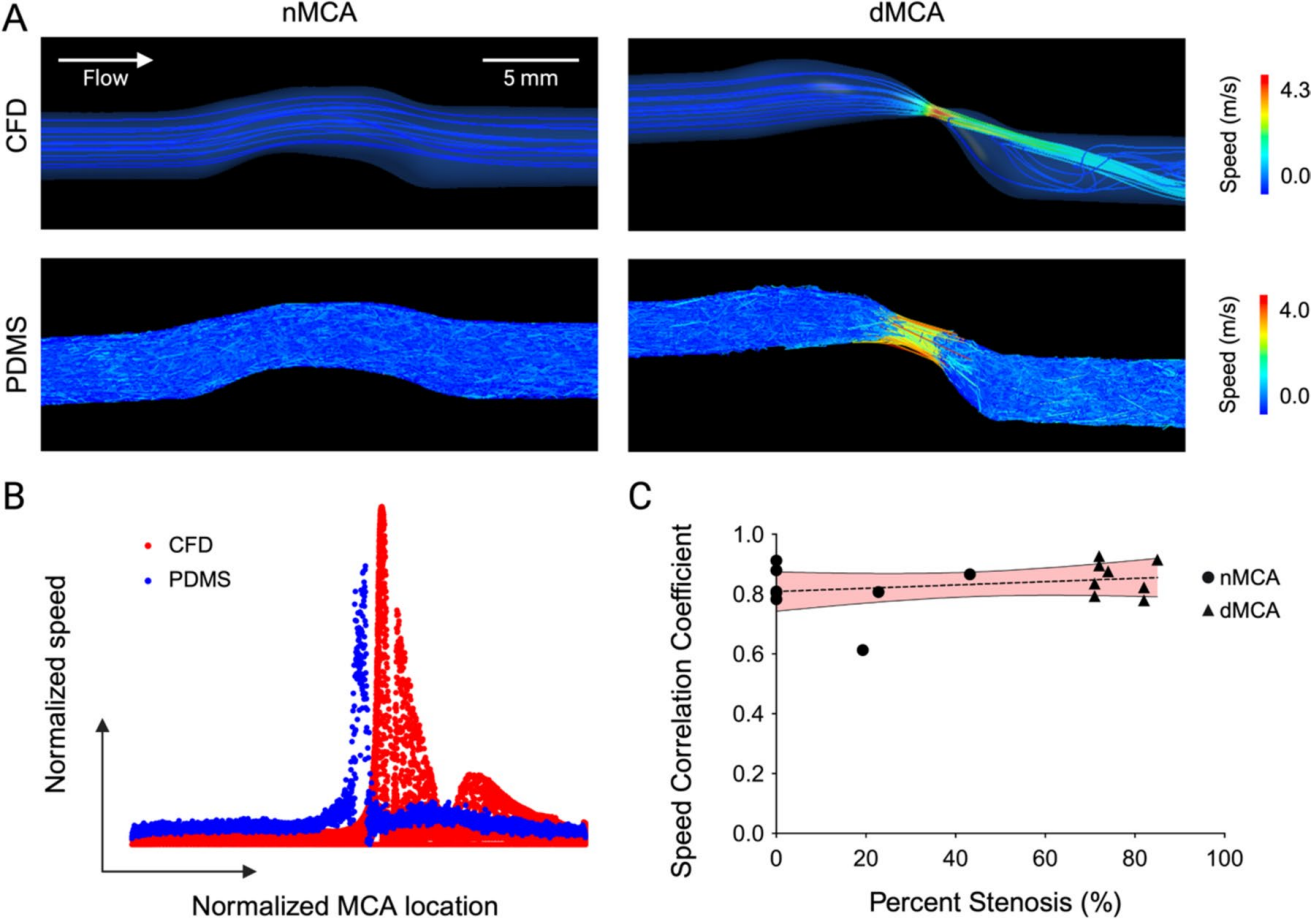
Validation of ICAD models by flow velocity comparisons. **A** Representative CFD velocity profiles and time-lapse particle flow studies on PDMS models generated from the same.stl files for one MCA case. **B** Representative plots of overlapping CFD (red) and PDMS (blue) normalized flow speed profiles as a function of location along the long axis of the vessel for the same stenotic dMCA arterial segment. **C** Plot of the speed correlation coefficient between corresponding CFD and PDMS models vs. percent stenosis (*n* = 16). The best-fit line is a simple linear regression (slope = 0.0006, *R*^2^ = 0.066, *p* = 0.338), and the error band represents 95% confidence intervals

To validate the use of sub-selected MCA arterial segments to identify novel biologic hallmarks of ICAD, we systematically compared flow velocity parameters measured by computational fluid dynamics and time-lapse particle flow. Using CFD, we obtained an average maximum speed of 3.32 m/s in the dMCA models and 0.34 m/s in the nMCA models (Fig. 3A). Using time-lapse particle flow studies in the PDMS models, we measured an average maximum speed of 3.98 m/s in the dMCA models (1.43 ± 0.26 × 10^3^ particles per model) and 2.32 m/s in the nMCA models (1.36 ± 0.11 × 10^3^ particles per model). The dynamics of these velocity profiles measured by CFD and particle flow were tightly associated across the pre-stenosis, intra-stenosis, and post-stenosis arterial locations (Fig. 3B). Comparing the flow speed profiles between predictive CFD measurements and experimental measurements in PDMS models, we observe an average correlation coefficient of 0.83 (*p* < 0.0001) across all modeled MCA segments (*n* = 16) (Fig. 3C). No relationship exists between velocity profile correlations and degree of vessel stenosis (slope = 0.0006, *R*^2^ = 0.066, *p* = 0.3379).

**Fig. 4:**
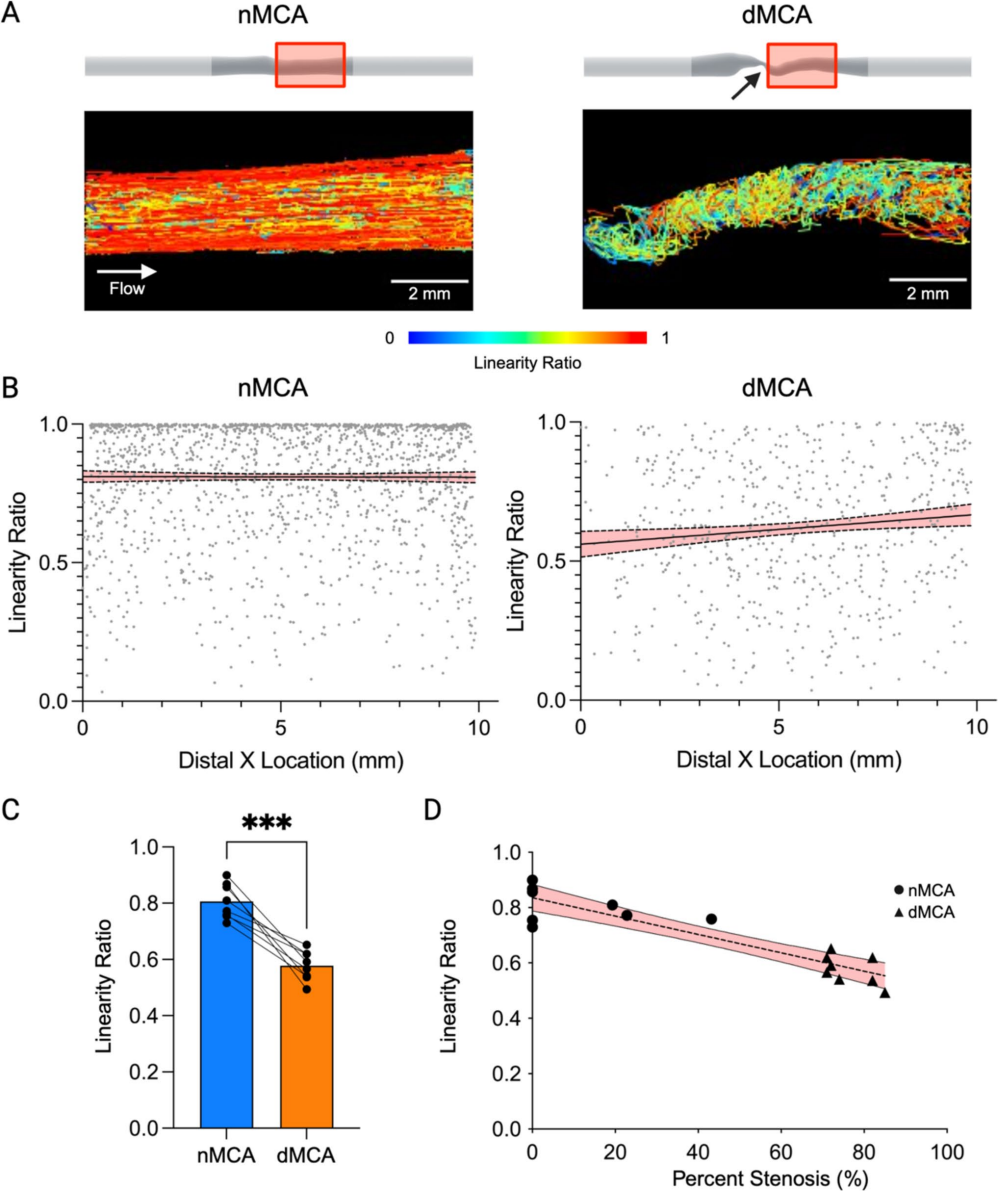
ICAD stenoses disrupt normal linear flow patterns. **A** Representative images of time-lapse particle flow studies in distal nMCA and dMCA segments. Red boxes indicate the location of distal ROI. Black arrow indicates the location of stenosis. **B** Plots of linearity ratio vs. vessel segment location (long axis). Best-fit lines are simple linear regressions (nMCA: slope = − 0.00026, *R*^2^ = 1.32 × 10^−5^, *p* = 0.884; dMCA: slope = 0.01069, *R*^2^ = 0.012, *p* = 0.006), and the error band represents 95% confidence intervals. **C** Mean linearity ratios in dMCA models (*n* = 8) compared to nMCA models (*n* = 8) (***p < 0.0001). **D** Best fit line is a simple linear regression (slope = − 0.0033, *R*^2^ = 0.828; *p* < 0.0001), and the error band represents the 95% confidence intervals

Particle flow data from MCA arterial segments demonstrated significant post-stenotic flow abnormalities (Fig. 4A). The linearity ratio of particle tracks measures the ratio of displacement of flow particles within the vessel segment across the total distance traveled. In the post-stenotic portion of the dMCA models, we observe disturbed flow with a markedly depressed linearity ratio within approximately 10 mm downstream of the stenosis (Fig. 4B). Linearity ratios of particle tracks after this immediate post-stenotic segment revert and gradually approach normal values like those in the nMCA models (data not shown). The mean linearity ratio in the post-stenotic region is significantly different between dMCA and nMCA models (0.58 vs. 0.81; *p* < 0.0001) (Fig. 4C). This change in post-stenotic flow measured by linearity ratio demonstrates an inverse linear relationship with percent vessel stenosis (slope = − 0.0033, *R*^2^ = 0.828; *p* < 0.0001) (Fig. 4D).

### Flow-Dependent and Stenosis-Dependent Endothelial Gene Expression Identified by sc-RNA-seq

**Fig. 5:**
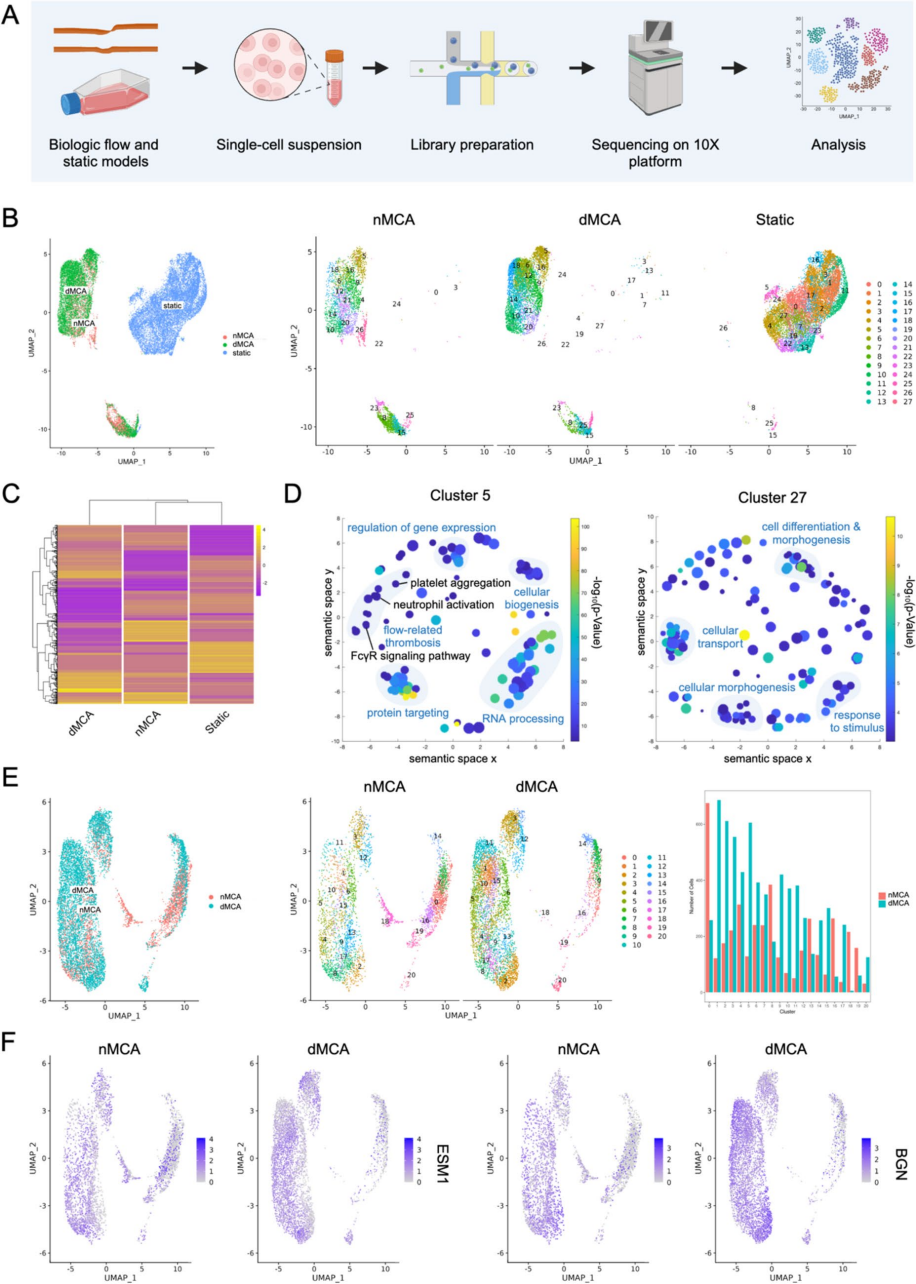
Flow-dependent and stenosis-dependent endothelial gene expression identified by sc-RNA-seq. **A** ScRNA-seq workflow for three conditions: nMCA, dMCA, and static culture. **B** Aggregate and single-sample UMAPs of nMCA, dMCA, and static models. **C** Heatmap representing logFC of genes (rows) in dMCA, nMCA, and static cell populations (columns). **D** Revigo plots of non-redundant gene ontology (GO) terms for subclusters 5 (flow-dependent) and 27 (non-flow dependent). Light blue regions indicate groupings of related GO terms. **E** Aggregate and single-sample UMAPs of nMCA and dMCA datasets. Cells per subcluster originating from the nMCA or dMCA model. **F** Feature plots of ESM1 and BGN in the nMCA and dMCA models

To identify different endothelial transcriptional profiles in 3D flow models and static cell culture, we analyzed mRNA transcripts of single cells from a dMCA model, its matched contralateral nMCA model, and a static culture flask using the 10 × Genomics platform (Fig. 5A). The cell viability for the nMCA, dMCA, and static samples was 63%, 63%, and 94%, respectively. All three samples passed standard cDNA and library analytical quality control checks. The total number of reads sequenced from the nMCA, dMCA, and static samples were 269 × 10^6^, 330 × 10^6^, and 317 × 10^6^, respectively.

After thresholding to remove 0.62% of total cells based on standard quality metrics of low transcript detection and high mitochondrial gene expression, we included a total of 4100 cells from the nMCA model, 6742 cells from the dMCA model, and 13,688 cells from the static model in our analysis. As demonstrated in the three-sample aggregate Uniform Manifold Approximation and Projection (UMAP), we identified a total of 28 transcriptionally distinct subclusters, eleven of which are associated almost exclusively with the 3D biologic flow models (5, 6, 9, 10, 12, 14, 15, 18, 20, 21, 26), four of which are associated almost exclusively with the static condition (2, 7, 13, 27), and 13 of which include a substantial proportion of cells from both flow and static samples (0, 1, 3, 4, 8, 11, 16, 17, 19, 22, 23, 24, 25) (Fig. 5B; Table 2). Using a heatmap visualization to compare the logFC of genes expressed across all conditions, we observed that there are distinct gene expression signatures for the dMCA, nMCA, and static conditions (Fig. 5C). To explore the different biologic processes enriched in flow versus static conditions, we created 2D plots of enriched gene ontology terms for subclusters 5 and 27, which are associated predominantly with flow and static populations, respectively (Fig. 5D). In the flow-associated subcluster 5, we identified five distinct groupings of GO terms related to regulation of gene expression, cellular biogenesis, flow-related thrombosis, protein targeting, and RNA processing. Within the flow-related thrombosis grouping, salient terms include enrichment for platelet aggregation, neutrophil activation, and FcγR signaling pathway as key GO terms that may be pertinent to the development of ICAD. In the static-associated subcluster 27, we found four groupings of GO terms related to cellular transport, cellular morphogenesis, response to stimulus, and cell differentiation and morphogenesis.

**Table 2:** Flow-responsive endothelial cell clusters

**Flow condition**	**Enriched endothelial cell clusters**	**Top Gene Ontology Terms**
Static culture	2	Positive regulation of cytokine production—GO:0001819 Cellular response to interleukin-4—GO:0071353
	7	Translational initiation—GO:0006413 Cotranslational protein targeting to membrane—GO:0006613
	13	Positive regulation of apoptotic signaling pathway—GO:2001235 Detoxification—GO:0098754
	27	Regulation of vasculature development—GO:1,901,342 Angiogenesis—GO:0001525 Negative regulation of cytokine production—GO:0001818 Platelet degranulation—GO:0002576 Cell surface receptor signaling pathway—GO:0007166 Cell–cell adhesion mediated by integrin—GO:0033631
Static and flow models	0	185 genes with no GO term enrichment
	1	DNA repair—GO:0006281 Regulation of histone H3-K9 methylation—GO:0051570
	3	Regulation of cyclin-dependent protein serine/threonine kinase activity—GO:0000079 Chromatin organization—GO:0006325
	4	Response to stimulus—GO:0050896 Regulation of localization—GO:0032879
	8	Regulation of cell communication—GO:0010646 Regulation of RNA metabolic process—GO:0051252
	11	Activation of innate immune response—GO:0002218 Lymphocyte activation involved in immune response—GO:0002285 Positive regulation of type I interferon production—GO:0032481
	16	Microtubule cytoskeleton organization—GO:0000226 Cell division—GO:0051301
	17	Response to topologically incorrect protein—GO:0035966 Cotranslational protein targeting to membrane—GO:0006613
	19	Leukocyte activation involved in immune response—GO:0002366 Neutrophil activation—GO:0042119
	22	Regulation of vasculature development—GO:1,901,342 Leukocyte activation—GO:0045321 Neutrophil activation—GO:0042119
	23	Negative regulation of blood circulation—GO:1,903,523 Negative regulation of vasoconstriction—GO:0045906 Positive regulation of lipopolysaccharide-mediated signaling pathway—GO:0031666
	24	Plasma cell differentiation—GO:0002317 Immune response-activating signaling pathway—GO:0002757 Cytokine-mediated signaling pathway—GO:0019221
	25	Mitochondrial translational elongation—GO:0070126 Regulation of hematopoietic progenitor cell differentiation—GO:1,901,532
3D flow models	5	Immune effector process—GO:0002252 Leukocyte activation involved in immune response—GO:0002366 Fc receptor mediated stimulatory signaling pathway—GO:0002431 Immune response-regulating cell surface receptor signaling pathway involved in phagocytosis—GO:0002433 MyD88-dependent toll-like receptor signaling pathway—GO:0002755
	6	Positive regulation of type II interferon production—GO:0032729 Positive regulation of interleukin-2 production—GO:0032743 Regulation of relaxation of muscle—GO:1,901,077
	9	Anatomical structure formation involved in morphogenesis—GO:0048646 Plasma membrane bounded cell projection assembly—GO:0120031
	10	Ossification—GO:0001503 RNA processing—GO:0006396 Protein-RNA complex organization—GO:0071826
	12	247 genes with no GO term enrichment
	14	Myeloid cell activation involved in immune response—GO:0002275 Leukocyte degranulation—GO:0043299 ncRNA processing—GO:0034470
	15	Adherens junction organization—GO:0034332 Positive regulation of cell migration—GO:0030335 Transmembrane receptor protein tyrosine kinase signaling pathway—GO:0007169
	18	432 genes with no GO term enrichment
	20	Immune effector process—GO:0002252 Myeloid leukocyte activation—GO:0002274 Leukocyte cell–cell adhesion—GO:0007159 Tumor necrosis factor-mediated signaling pathway—GO:0033209 NIK/NF-kappaB signaling—GO:0038061 Antigen processing and presentation of exogenous peptide antigen via MHC class I—GO:0042590
	21	Immune effector process—GO:0002252 Leukocyte activation—GO:0045321
	26	Response to extracellular stimulus—GO:0009991 Regulation of interleukin-4 production—GO:0032673 Apoptotic signaling pathway—GO:0097190

In comparison of the nMCA and dMCA 3D flow samples separate from the static sample, we detected 21 subclusters with unique transcriptional profiles as identified in the two-sample aggregate UMAP (Fig. 5E). All subclusters contained cells from both samples, but six subclusters had a greater proportion of cells from the nMCA sample (0, 8, 13, 16, 18, and 19) while the other 15 subclusters had a greater proportion of cells from the dMCA sample (1, 2, 3, 4, 5, 6, 7, 9, 10, 11, 12, 14, 15, 17, 20). Transcriptional expression levels of the canonical endothelial cell marker endothelial specific molecule-1 (ESM-1) is equal across nMCA and dMCA cell subclusters. Comparatively, the expression levels of the biglycan (BGN) gene implicated in atherosclerosis are enriched in dMCA-specific cell subclusters, including subclusters 2, 5, 11, and 20 (Fig. 5F).

### Aberrant Flow in a Stenotic MCA Model Drives Morphological Changes in Endothelial Cells

**Fig. 6:**
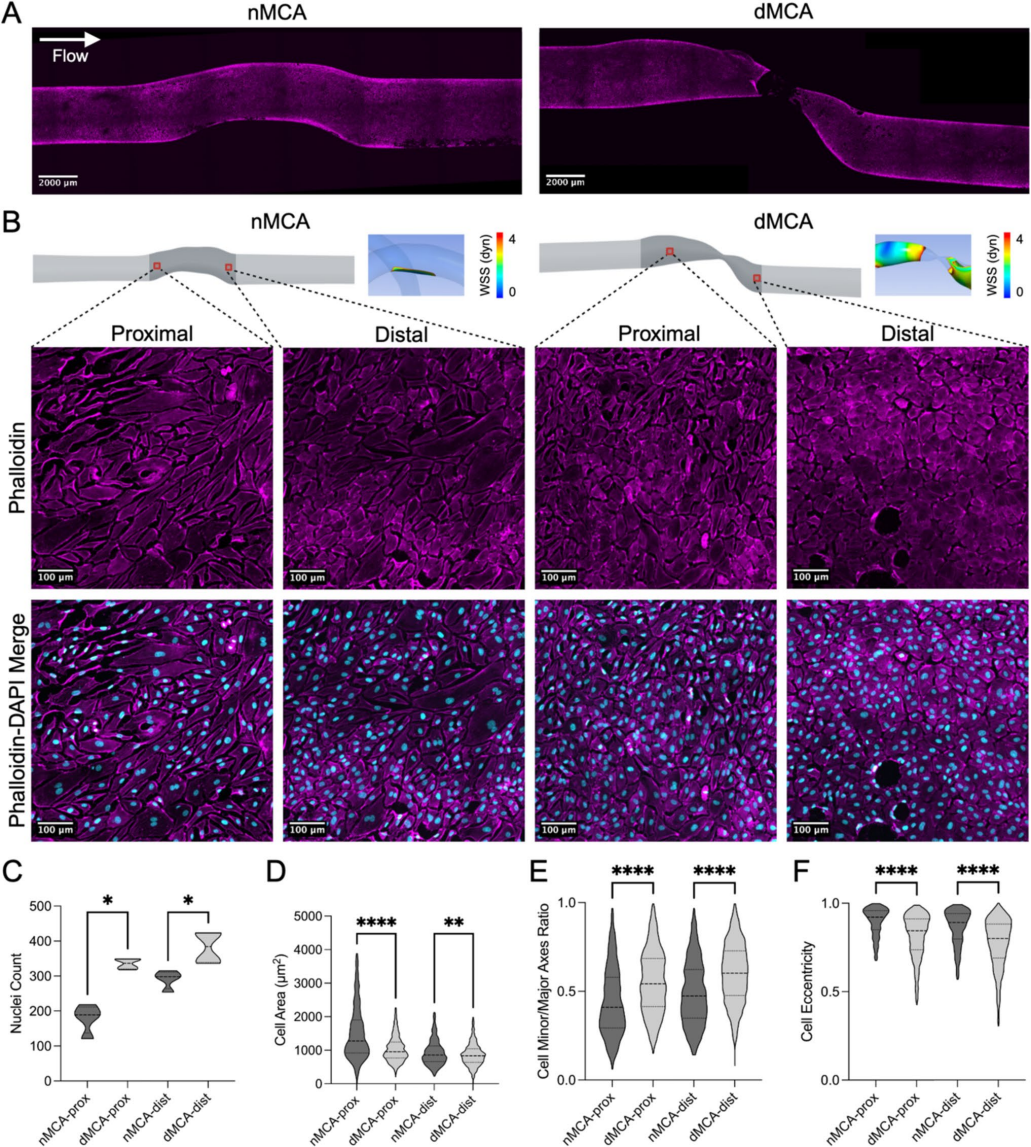
Aberrant flow in a stenotic MCA model drives morphological changes in endothelial cells. **A** Tile scan images of phalloidin-labeled cells from matched nMCA and dMCA 3D biologic flow models (scale bars = 2000 µm). **B** Representative images in distal and proximal ROIs in the nMCA and dMCA models. Red boxes indicate proximal and distal ROIs (top left) with CFD WSS profiles of native MCAs in the top right. **C** Violin plot of nuclei counts in proximal (*p = 0.0286) and distal (*p = 0.0286) nMCA and dMCA segments (*n* = 4/condition). **D** Violin plot of cell area in proximal (****p < 0.0001) and distal (**p = 0.0013) nMCA and dMCA segments. **E** Violin plot of cell minor/major axes ratio in proximal (****p < 0.0001) and distal (****p < 0.0001) nMCA and dMCA segments. **F** Violin plot of cell eccentricity in proximal (****p < 0.0001) and distal (****p < 0.0001) nMCA and dMCA segments. Data represent quantitation of 533 cells in proximal nMCA, 977 cells in distal nMCA, 1243 cells in proximal dMCA, and 1530 cells in distal dMCA

To identify regional changes in endothelial cell number and morphology, we stained for F-actin filaments and used confocal microscopy to visualize the entire length of a dMCA stenosis model and its associated contralateral nMCA model (Fig. 6A). We focused on one proximal region and one distal region in each of the nMCA and dMCA models (Fig. 6B). In the dMCA model, both the proximal and distal regions of interest are exposed to low wall shear stress values (< 4 dynes/cm^2^) whereas the proximal and distal regions of interest in the nMCA model are exposed to wall shear stress values within a normal range (> 4 dynes/cm^2^) as determined by CFD (Fig. 6B). In terms of cell number, there was significantly greater nuclei count in the proximal (335.0 ± 14.94 vs. 179.3 ± 41.24, *p* = 0.0286) and distal (382.0 ± 46.45 vs. 291.3 ± 26.15, *p* = 0.0286) regions of the dMCA model compared to the associated regions in the nMCA model (Fig. 6C). Conversely, we observed that cells in the dMCA model had significantly smaller areas in the proximal (1142 ± 621.1 mm^2^ vs. 2026 ± 2275 mm^2^, *p* < 0.0001) and distal (894.4 ± 403.6 mm^2^ vs. 1071 ± 735.9 mm^2^, *p* = 0.0013) regions relative to cells in these regions of the nMCA model (Fig. 6D). We also found that cells in the dMCA model had higher minor/major axes ratios in the proximal (0.55 ± 0.18 vs. 0.44 ± 0.19, *p* < 0.0001) and distal (0.60 ± 0.17 vs. 0.49 ± 0.18, *p* < 0.0001) regions compared to cells in these regions in the nMCA model (Fig. 6E). Finally, we observed that cells in the dMCA model demonstrated a lower degree of eccentricity in the proximal (0.80 ± 0.14 vs. 0.87 ± 0.12, *p* < 0.0001) and distal (0.77 ± 0.14 vs. 0.84 ± 0.13, *p* < 0.0001) regions than cells in the associated regions in the nMCA model (Fig. 6F).

## Discussion

There is an urgent need to identify the biologic underpinning of stroke caused by ICAD. Here, we introduce and validate a novel, precision 3D cerebrovascular modeling approach for ICAD lesions that draws on highly relevant neuroimaging studies and incorporates endothelial cell biology to investigate the relationship between aberrant hemodynamics and biologic pathways relevant to ICAD. By demonstrating a tight relationship between predicted CFD and observed particle flow velocities across multiple MCA vessel segments, we validate the reproducibility of this model system for studying ICAD lesions. Using single-cell RNA-seq and microscopy of endothelial cell morphology, we establish a link between the abnormal flow forces induced in ICAD lesions and the biologic consequences on endothelial cells. Together, these findings validate this approach as a novel model of ICAD useful for mechanistic studies of biologic pathways in ICAD as well as drug discovery.

In developing a combined in silico*-*in vitro modeling approach of intracranial stenosis, we first sought to establish that the differences in anatomical structure and flow dynamics between CFD and PDMS models of the same patient-specific arterial segments were negligible. Since each corresponding pair of CFD and PDMS models originate from the same.stl file, both models have identical 3D structures and thus can be directly compared at any given spatial location throughout the vessel segment. Furthermore, the strong correlation between the flow speed profiles in corresponding CFD and PDMS models validates that predictive CFD correlates with measured flow-based phenomena in 3D biologic flow models. Additionally, the fact that there is no relationship between the correlation coefficient and stenosis diameter across a range of unique cases implies that any given pair of CFD and PDMS models representing the same vessel segment should have similar flow speed profiles, regardless of stenosis severity. Taken together, these findings indicate that CFD and PDMS models of the same arterial segment are highly comparable in terms of 3D structure and fluid dynamics, which in turn justifies subsequent correlations of hemodynamic values like wall shear stress from CFD models and biologic findings from the associated 3D biologic flow models.

Our results also show that the pattern of flow is significantly disturbed in the post-stenotic region of the dMCA PDMS models relative to the associated regions of nMCA models, which provides direct evidence that the fluid dynamics in these models are comparable to those in diseased arteries in ICAD patients. The presence of disturbed flow in the dMCA models is a critical feature of our ICAD modeling approach as disruption of laminar flow is a central mechanism suggested to underlie ICAD lesion progression and subsequent stroke [12–15]. Furthermore, our results reveal an inverse linear relationship between the linearity ratio in the post-stenotic region and stenosis severity, indicating that more severe stenoses give rise to greater degrees of disturbed flow in the post-stenotic region.

The application of scRNA-seq on cells from a pair of dMCA and nMCA 3D biologic flow models demonstrates the feasibility of this technique for investigating the biologic effects of ICAD-related flow on endothelial cells. Interestingly, the identification of 28 transcriptionally distinct subclusters in our aggregate analysis of cells from the dMCA, nMCA, and static samples indicates that there is a moderate degree of biodiversity across samples, even though all three samples are comprised only of endothelial cells. Notably, cells from the two 3D biologic flow models share a more similar transcriptional profile than cells grown under static conditions as might be suspected. Somewhat surprisingly given that the MCA flow models are highly similar except for the stenosis region, we nonetheless can reliably use scRNA-seq to identify multiple different endothelial cell subclusters by transcriptional profiling, suggesting that there is significant endothelial cell heterogeneity within ICAD lesions.

The flow-related thrombosis grouping of GO terms in subcluster 5 highlights a variety of interesting pathways (platelet aggregation, neutrophil activation, FcγR signaling pathway) relevant to vascular occlusion and stroke. Upregulation of the FcγR signaling pathway is a particularly interesting finding as prior work suggests there may be a causative relationship between endothelial shear stress and platelet activation via platelet FcγRIIa expression in ICAD [12]. Moreover, the enrichment of these GO terms in a single flow-related subcluster demonstrates how the application of scRNA-seq enabled the identification of a smaller subpopulation of cells implicated in thrombosis that may not have been identifiable via traditional bulk RNA sequencing. This finding underscores the fact that utilizing single-cell analyses in our biologic models of ICAD is not only possible but necessary to study the nuanced contribution of flow to ICAD development and stroke.

Comparing the data from the dMCA and nMCA flow samples only, we identified ESM-1 as a notable gene among the list of conserved cell markers for subcluster 9 in Fig. 5E. In the feature plots of ESM-1 in Fig. 5F, we observed that ESM-1 was upregulated relatively equally in both the nMCA and dMCA plots compared to its average expression across the human genome, which is consistent with previous data establishing the gene as a canonical marker of endothelial cells [16, 17]. Additionally, BGN stood out to us as an interesting gene among the list of conserved cell markers for subcluster 10, which has a substantially high proportion of cells from the dMCA condition. Although feature plots of the dMCA and nMCA samples both demonstrate upregulation of BGN, the number of cells with a high expression level of BGN is substantially greater in the dMCA sample than in the nMCA sample. Considering that BGN overexpression increases lipid retention and atherosclerosis development [18–21], the finding that BGN transcription is upregulated in the dMCA cell population suggests that BGN may play an important role in the development of ICAD and potentially serve as a novel drug target for the disease.

In addition, we have also successfully employed cell labeling and confocal imaging to visualize cells in our 3D biologic flow models at the level of single-cell resolution. The capacity to image individual endothelial cells in our models enables us to identify regional differences in cell number, morphology, and protein expression, which may be useful for precise localization of where specific pathogenic changes take place in the diseased artery. Here, we focused on easily observable changes in endothelial cell number and morphology in the proximal and distal regions of paired nMCA and dMCA models from the same patient case. More complex analyses including target protein-specific intensity would also be feasible using this approach. Since both proximal and distal regions in the dMCA model are exposed to lower WSS values and larger WSS gradients due to their proximity to the stenosis, this finding is consistent with prior literature that reports an increased rate of DNA synthesis [22] and proliferation [23, 24] in regions of low WSS and large WSS gradients. Morphological changes such as cellular eccentricity and minor/major axes ratio that were distinct in the dMCA model are consistent with previous work demonstrating that endothelial cells take on a rounded shape in regions of low WSS and become more elongated in regions of high WSS [22, 25]. The fact that these findings replicate known flow-dependent trends in endothelial cell number and morphology validates that our ICAD modeling approach is a reliable tool to investigate endothelial changes in response to different patterns of flow.

As with any model of complex disease, ours is not without its limitations. Case selection bias represents one limitation as each eligible case must have both CTA and DSA imaging, and we cannot model cases that have excessive tortuosity, multiple symptomatic lesions, and/or lesions that are too close to an arterial branching point. The impact of downstream branches on flow gradients cannot be easily studied in this model. Moreover, presently, the model incorporates only the endothelial response to flow phenomena and cannot model the intercellular interactions that may drive atherosclerotic biology. There are also several technical limitations related to measurements within the model. Camera acquisition speeds were too slow to detect particles in the high-speed stenotic region, so we enabled frame jumping and gap closing to account for frames when the particles were passing through the stenosis. Additionally, since the flow speed data in the PDMS models was acquired as 2D videos, we had to collapse the *z*-axis of 3D flow speed data from the CFD models to compare both datasets. We were also unable to perfectly align the normalized MCA location data from each corresponding pair of CFD and PDMS models when calculating the flow speed correlation coefficient. Despite these limitations, we still obtained a high average correlation coefficient for the flow speed profile between corresponding CFD and PDMS models. From a biologic standpoint, we made a pragmatic choice to use HUVECs for endothelialization. While these cells display recognized responses to flow forces including shear stress, future studies may consider the use of human carotid artery cells that may be closer to the native response of intracranial vasculature. Uniform endothelialization across and within ICAD models is not to be expected, and the cell count alone should not be presumed to be a measure of proliferation. A key limitation of the sc-RNA seq experiment is our inability to determine the spatial origins of identified subclusters within the model. However, confirmatory studies such as RNA in situ hybridization or protein labeling by immunofluorescence can be used to locate where transcriptional changes identified by sc-RNA seq occur. Finally, the macroscale of the MCA model makes it challenging to image on most confocal setups, and resolution quality diminishes substantially with depth into the model. To address this limitation, we can acquire multiple images of the model rotated in different orientations depending on the size and number of the areas of interest. Alternatively, a light sheet microcopy can be considered.

## Conclusion

In this study, we introduce and validate a novel model for ICAD that can address key questions in the biologic pathways relevant to ICAD. Application of this model platform to other key vessel segments affected by ICAD including the internal carotid, basilar, and vertebral arteries to identify commonalities and differences in flow and endothelial cell responses to ICAD lesions in different portions of the cerebral circulation is critical to establishing shared mechanisms. The identification of biologic pathways that respond to the unique hemodynamic forces created by ICAD lesions compared to atherosclerosis in other tissue beds will be essential for the development of new therapeutics to reduce the high risk of stroke associated with ICAD.

## Data Availability

Study related data is shared via Open Science Framework: https://osf.io/zm3ep.
